# Intrinsic Strand-Incision Activity of Human UNG: Implications for Nick Generation in Immunoglobulin Gene Diversification

**DOI:** 10.3389/fimmu.2021.762032

**Published:** 2021-12-22

**Authors:** Marina Alexeeva, Marivi Nabong Moen, Xiang Ming Xu, Anette Rasmussen, Ingar Leiros, Finn Kirpekar, Arne Klungland, Lene Alsøe, Hilde Nilsen, Svein Bjelland

**Affiliations:** ^1^Department of Chemistry, Bioscience and Environmental Technology, University of Stavanger, Stavanger, Norway; ^2^Department of Microbiology, Oslo University Hospital, Oslo, Norway; ^3^Department of Biochemistry and Molecular Biology, University of Southern Denmark, Odense, Denmark; ^4^Department of Chemistry, University of Tromsø-The Arctic University of Norway, Tromsø, Norway; ^5^Department of Clinical Molecular Biology, University of Oslo, Oslo, Norway; ^6^Section of Clinical Molecular Biology, Akershus University Hospital, Lørenskog, Norway

**Keywords:** human UNG, class switch recombination, somatic hypermutation, immunoglobulin diversification, cytosine deamination

## Abstract

Uracil arises in cellular DNA by cytosine (C) deamination and erroneous replicative incorporation of deoxyuridine monophosphate opposite adenine. The former generates C → thymine transition mutations if uracil is not removed by uracil-DNA glycosylase (UDG) and replaced by C by the base excision repair (BER) pathway. The primary human UDG is hUNG. During immunoglobulin gene diversification in activated B cells, targeted cytosine deamination by activation-induced cytidine deaminase followed by uracil excision by hUNG is important for class switch recombination (CSR) and somatic hypermutation by providing the substrate for DNA double-strand breaks and mutagenesis, respectively. However, considerable uncertainty remains regarding the mechanisms leading to DNA incision following uracil excision: based on the general BER scheme, apurinic/apyrimidinic (AP) endonuclease (APE1 and/or APE2) is believed to generate the strand break by incising the AP site generated by hUNG. We report here that hUNG may incise the DNA backbone subsequent to uracil excision resulting in a 3´-α,β-unsaturated aldehyde designated uracil-DNA incision product (UIP), and a 5´-phosphate. The formation of UIP accords with an elimination (E2) reaction where deprotonation of C2´ occurs *via* the formation of a C1´ enolate intermediate. UIP is removed from the 3´-end by hAPE1. This shows that the first two steps in uracil BER can be performed by hUNG, which might explain the significant residual CSR activity in cells deficient in APE1 and APE2.

## Introduction

Hydrolytic deamination of cytosine (C) to uracil (U) in DNA is a frequent event in all including human cells. Because uracil instructs insertion of adenine (A), uracil has to be efficiently removed before replication to avoid formation of C → thymine (T) transition mutations (1, 2). Consequently, all cells need a DNA repair system for uracil (3). Most cells employ the base excision repair (BER) pathway (4, 5) initiated by a uracil-DNA glycosylase (UDG; EC 3.2.2.27). The level of C → T mutations in a cell or organism is increased by one order of magnitude if the UDG function(s) is disrupted (3). If deoxyuridine triphosphate (dUTP) escapes degradation by dUTPase (6), uracil may also be misincorporated in DNA in place of thymine. The UDG superfamily (7) comprises five families exhibiting similar architecture and organization of the enzyme active site. Human cells contain one family 1 (uracil-DNA *N*-glycosidase; hUNG), one family 2 (thymine-DNA glycosylase; hTDG), and one family 3 (human single-strand-selective mono-functional UDG; hSMUG1) UDG. The major and most effective UDG for removal of uracil from nuclear DNA is hUNG2, while hUNG1 is the mitochondrial splice variant (8). hUNG is removing both deaminated cytosine and misincorporated uracil, with high activity for uracil in single-stranded (ss) DNA, which is enriched in replicating cells. In contrast, the human family 3 UDG hSMUG1 (9) is important for repair of uracil in the general genome (10) but also removes oxidized pyrimidines (11, 12). hTDG can remove T or U when base-paired with G but appears to have a main role in active DNA demethylation (13–15). hUNG exhibits a tight active site that is specific for uracil (16). Hitherto, family 1–3 UDGs have been classified as mono-functional DNA glycosylases only able to excise the damaged base. This contrasts with the bi-functional DNA glycosylases that exhibit additional lyase activity carrying out a β- or β/δ-elimination reaction to incise the apurinic/apyrimidinic (AP) site, although such incision is believed to predominantly being accomplished by human AP endonuclease 1 (hAPE1) (17–19) *in vivo*, which is also able to process an AP ribonucleoside embedded in DNA (20). The 3´-deoxyribose phosphate (dRP) and 3´-α,β-unsaturated aldehyde remnants after the β-elimination reaction are also removed by the 3´-phosphodiesterase function of hAPE1, whereas the 3´-phosphate left after the β/δ-elimination reaction is removed by the human polynucleotide kinase phosphatase (1, 21). The BER pathway is completed by the sequential action of DNA polymerase β (22), which also removes the 5´-dRP by its lyase function if hAPE1 incised the AP site, and DNA ligase (1, 2, 5).

In higher vertebrates, UNG is important for immunoglobulin gene diversification (23). In activated B cells, activation-induced cytidine deaminase (AID) (24, 25) deaminates cytosine to uracil (26) and thereby initiates immunoglobulin gene diversification through somatic hypermutation (SHM) (27, 28) and class switch recombination (CSR) [see reference (29) for review]. CSR requires a number of enzymes and pathways. One major processing pathway involves uracil excision by UNG. This role of UNG is supported by severely reduced CSR in *Ung*-deficient mice (30–34). It is also supported by mutations in the *UNG* gene leading to development of hyper-IgM syndrome type 5 (35–37), an immunodeficiency syndrome caused by defective CSR. AID-induced uracil residues must be converted into DNA double strand breaks (DSB) to allow CSR because DSB serve as substrates for the DNA rearrangements mediated by non-homologous end-joining. According to the classical CSR model, this implicates that uracil excision by UNG is followed by incision at AP sites by AP endonuclease to generate a nicked substrate that can be further processed into DSB (23). However, our recent discovery that hSMUG1 nicks the AP site after uracil removal (38) encouraged us to investigate whether hUNG does the same under the same experimental conditions.

## Materials and Methods

### Oligonucleotide Substrates

The following polydeoxynucleotides with deoxyuridine monophosphate (dUMP) and a 5´-Cy3 fluorophore, as indicated and protected by phosphorothioate (four bonds) at each end (from Sigma-Aldrich or Eurofins MWG), were annealed to equimolar amounts of a complementary strand with G opposite U as described (38): [Cy3] 5´-TAGACATTGCCCTCGAGGTAUCATGGATCCGATTTCGACCTCAAACCTAGACGAATTCCG-3´ [60 nucleotides (nt); substrate 1]; [Cy3] 5´-CCCTCGAGGTAUCATGGATCCGATCG-3´ [26 nt; substrate 2, used unlabeled and without end-protection in the matrix-assisted laser desorption/ionization time-of-flight mass spectrometric (MALDI-TOF-MS) analyses]; [Cy3] 5´-CCCTCGAGGTAUCATGGATCCGATCGATCCGATTTCGACCTCAAACCTAGACGAATTCCG-3´ (60 nt; substrate 3).

### Enzymes

hUNG protein (hUNGΔ84 with/without His-tag) (39, 40) was provided by B. Kavli and G. Slupphaug. EcUng was obtained from NEB, Fermentas and Trevigen; EcNfo was obtained from Fermentas; EcFpg, EcNth, hOGG1, and hAPE1 were obtained from NEB. hUNGΔ84 mutant proteins were purified as described (41) with the following minor modifications: bacteria were grown in 0.5 or 1 l of Terrific Broth (TB) medium containing 100 μg/ml ampicillin and lysed by sonication; proteins were purified in three steps by anion and cation exchange and size exclusion chromatography using HiTrap Q (5 ml), HiTrap SP (5 ml) and HiLoad Superdex 75 16/600 columns, respectively, in that order. The amino acid replacements were confirmed by Q-Exactive [liquid chromatography (LC)-MS/MS] and Denovo sequencing (see **Supplementary Figure 6**).

### Assays for Incision of U-DNA

**Figure 1:**
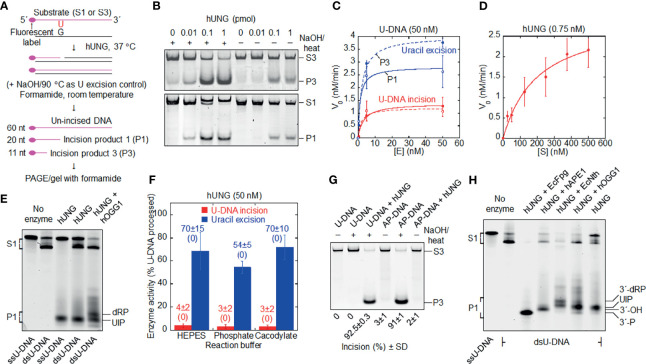
Formation of uracil-DNA incision product (UIP) by hUNG. **(A)** DNA substrate and base excision assay. Either substrate 1 (S1) or substrate 3 (S3; the base sequences of their labeled strands are presented in Materials and Methods) was exposed to hUNG, resulting in incision product 1 (P1) or incision product 3 (P3), respectively. hUNG (1 pmol) was incubated 10 min with DNA substrate (1 pmol) at 37°C in 45 mM HEPES [4-(2-hydroxyethyl)-1-piperazineethane-sulfonic acid]–KOH, pH 7.8, 0.4 mM EDTA, 1 mM DTT, 70 mM KCl, and 2% (v*/*v) glycerol (reaction buffer; final volume, 20 µl), if not otherwise stated. **(B)** Protein dependence of dsU-DNA incision (red) and uracil excision (blue). PAGE was performed at 100 V for 30 min (upper panel) or 50 min (lower panel) using a 12% (w/v) gel, which contained 3% (v/v) formamide, where **(C)** presents the three to four independent measurements performed. **(D)** Incision of dsU-DNA by hUNG follows *Michaelis* **–***Menten* kinetics. hUNG (0.015 pmol) was incubated with an increasing concentration of S1 for 20 min, where the other experimental details are the same as in **(B)** [except that a 20% (w/v) gel was used]; each value represents the average of 7 independent measurements. **(E)** Cleavage of ss- and dsU-DNA by hUNG. S1 or its labeled strand was incubated with hUNG alone for 20 min (third and fourth lane), which in one case was followed by incubation together with hOGG1 (3 pmol) for additional 30 min (fifth lane). PAGE was performed at 300 V for 7 h using a 20% (w/v) gel containing 7 M urea. **(F)** Incision of dsU-DNA by hUNG in different buffers. S1 was incubated with and without hUNG in reaction buffer (HEPES), 45 mM sodium cacodylate, or 45 mM potassium phosphate, with the same pH and additions as for reaction buffer (see **Supplementary Figure 1** for details). In each case, five independent measurements were performed. Each value represents the average ± SD, where control value without enzyme is shown in parenthesis. **(G)** No cleavage of dsAP-DNA (U replaced by AP site) by hUNG. dsU-DNA (S3, 1 pmol) was converted to dsAP-DNA by incubation with EcUng (2 pmol) at 37°C for 10 min. AP- or U-DNA was incubated with and without hUNG. PAGE was performed at 100 V for 1 h using a 12% (w/v) gel, which contained 3% (v/v) formamide. The inability of the NaOH/heat treatment to cleave U-DNA as opposed to AP-DNA verified the integrity of the former as well as the nature of the latter. The complete cleavage of U-DNA by the NaOH/heat treatment following hUNG exposure verified active enzyme. It should be noted that we always detected UIP in our samples of AP-DNA, which was difficult to avoid since we routinely used EcUng for its preparation. In the AP-DNA sample presented in the figure, we succeeded to wash away most UIP during the two precipitation steps employed. Three independent experiments with virtually the same result were performed. **(H)** Indirect identification of UIP by its electrophoretic mobility compared to that of characterized enzymes. hUNG (1 pmol) was incubated with S1 (1 pmol); either alone for 20 min (seventh lane), together with EcFpg (4 pmol) for 30 min (third lane), alone for 20 min and thereafter together with hAPE1 (0.45 pmol) for an additional 30 min (fourth lane), together with EcNth (1 pmol) for 30 min (fifth lane), or alone for 20 min and thereafter together with hOGG1 (4 pmol) for an additional 30 min (sixth lane). Different incision products were separated from un-incised DNA by PAGE at 300 V for 7 h using a 20% (w/v) gel containing 7 M urea. The experiment with the arrangement presented was performed three times. More than 10 additional experiments were performed with other arrangements to indicate the 3´ incision product. dRP, deoxyribose phosphate; nt, nucleotides; P, phosphate.

Proteins were incubated with substrate 1, 2 or 3, or AP-DNA, at 37°C in 45 mM HEPES [4-(2-hydroxyethyl)-1-piperazineethanesulfonic acid]-KOH, pH 7.8, 0.4 mM ethylenediaminetetraacetic acid (EDTA), 1 mM dithiothreitol (DTT), 70 mM KCl, and 2% (v/v) glycerol (reaction buffer) (final volume, 20 μl). Reactions were terminated by the addition of 20 mM EDTA, 0.5% (w/v) sodium dodecyl sulfate (SDS), and proteinase K (190 µg/ml) followed by precipitation of DNA with 96% ethanol containing 0.1 M sodium acetate supplemented with 16 µg tRNA and solubilization in 10 µl water (38). To eliminate non-enzymatic cleavage of AP sites, the samples (10 µl; DNA dissolved in water) were treated at room temperature by the addition of loading solution with 80% (v/v) formamide, 1 mM EDTA, and 0.05% xylene cyanol (10 µl). The samples were subjected to polyacrylamide gel electrophoresis (PAGE) without delay (see **Figure 1A**). The gel [12–20% (w/v) polyacrylamide] contained 3% (v/v) formamide (see **Figure 1B**). In the experiments carried out to determine the relative migration of the different 3´-end products, the gel [20% (w/v) polyacrylamide] contained 7 M urea (see **Figure 1H**). DNA glycosylase activity was determined in parallel by NaOH-mediated (10 min at 90°C with 0.1 M final concentration) incision of AP sites (38). Visualization and quantification were performed by fluorescence or phosphor imaging analysis using ImageQuant Software (Molecular Dynamics Inc.). The graphs were drawn using KaleidaGraph version 4.1.0 (Synergy Software).

### MALDI-TOF-MS Analysis of Uracil-DNA Digested by hUNG in Normal Water or H_2_^18^O

Reaction mixtures with hUNG (28 pmol) and unlabeled substrate 2 (normal H_2_^16^O experiments, 10 pmol; H_2_^18^O experiments, 20 pmol) were incubated in 20 mM Tris-HCl, pH 8.0, 1 mM DTT, 1 mM EDTA, 70 mM KCl at 37°C for 30 min (normal H_2_^16^O experiments; final volume, 20 μl), or 1 h (H_2_^18^O experiments; final volume, 10 μl). Control incubations were performed with EcUng (0.76 pmol) plus either hOGG1 (263 pmol), EcNth (8.7 pmol), EcFpg (17 pmol), or EcNfo (0.03 pmol) to compare the hUNG-generated 3´-end product with those of characterized enzymes. MALDI-TOF-MS analysis of reaction products was carried out as described (42). Substrate DNA was evaporated using vacuum centrifugation followed by re-suspension in H_2_^18^O (Aldrich, Product No. 329878; 20 µl). The ^18^O-labeling of the enzymatic products was performed by dissolving them in H_2_^18^O followed by incubation at 4°C overnight. The MS was performed as above, but with H_2_^18^O replacing H_2_^16^O in every step. DNA was precipitated with 96% ethanol, 1 M ammonium acetate, and 0.1 µg/µl glycogen followed by incubation at ‒20°C overnight (for some experiments, precipitation was performed as in the experiments using PAGE as described above). DNA pellet was collected by centrifugation at 13,000 rpm for 30 min at 4°C.

### Structural Analysis

The crystal structures of hUNG complexes with dsDNA containing the substrate analog pseudouracil (PDN1emh) (43) or an AP site (PDB2ssp) (44) were manually inspected and visualized using PyMOL (The PyMOL Molecular Graphics System, Version 1.5.0.4 Schrödinger, LLC).

## Results

### hUNG Can Cleave the AP Site Following Uracil Excision

When two uracil-containing DNA substrates (**Figure 1A**; substrate 1 and 3) were treated with hUNG, we observed hUNG-dependent cleavage of U-DNA at the lesion site (**Figure 1B**). DNA was denatured at room temperature to eliminate non-enzymatic AP site cleavage (38) and uracil excision activity was determined in parallel where necessary. We also observed that the incision level was remarkably similar for the two substrates (**Figure 1C**). Kinetic analysis of the U-DNA incision activity by hUNG indicated *Michaelis* **–***Menten behavior* (**Figure 1D**), the analysis giving a *K_m_* of 200 nM, a *k_cat_* of 4 min^-1^, and a *k_cat_/K_m_* of 0.02 min^-1^ nM^-1^ (**Table 1**). We additionally observed that hUNG incises ss and double-stranded (ds) U-DNA at similar efficiencies (**Figure 1E**; ssU-DNA is the labeled strand of substrate 1). This differs from AP lyases that exhibit low activity for ssDNA (1), largely excluding the possibility that enzymes other than UNG are involved. It is important to note that we always employed reaction conditions without Mg^2+^ and with EDTA added, to inhibit possible contaminating AP endonuclease activity (1), in spite of the fact that UDGs are stimulated by Mg^2+^ ions (39). Moreover, the presence of amines in the (HEPES) reaction buffer may lead to cleavage of AP sites in DNA *via* a β-elimination reaction (45), contributing to a false U-DNA incision activity. To investigate this possibility, we compared hUNG activity in HEPES and sodium cacodylate buffer in parallel experiments using otherwise identical conditions. The results showed no significant difference in incision activity between these two reaction buffers, which largely excludes possible artifacts related to reaction buffer composition (**Figure 1F** and **Supplementary Figure 1**). AP incision activity was coupled to uracil excision because no significant activity was detected at an AP site at the same place in DNA (**Figure 1G**).

**Table 1:** Kinetic parameters of the hUNG-catalyzed uracil-DNA incision activity.

Enzyme	*K_m_* (nM)	*k_cat_* (min^−1^)	*k_cat_/K_m_* (min^−1^ nM^−1^)
hUNG	200 ± 60	4.0 ± 0.5	0.020

These values were determined from the graph (**Figure 1D**) that shows the best fit (R = 0.989) to Michaelis**–**Menten behavior, calculated using KaleidaGraph version 4.1.0 (Synergy Software).

### Indirect Identification of the hUNG 5´ Incision Product as a 3´-α,β-Unsaturated Aldehyde

The mobility of the 5´-labeled incision fragment was compared with fragments generated by other bi-functional DNA glycosylases and AP endonucleases, i.e., U-DNA (substrate 1) was pretreated with family 1 UDG of *Escherichia coli* (EcUng) to convert uracil into an AP site followed by treatment with either (a) *E. coli* endonuclease III (EcNth) to define a 3´-dRP formed by β-elimination (46), (b) hAPE1 to define a 3´-OH (47), (c) *E. coli* formamidopyrimidine-DNA glycosylase (EcFpg) (48) to define a 3´-phosphate formed by β/δ-elimination (δ-product), or (d) human 8-oxoguanine-DNA glycosylase (hOGG1) to define the 3´-α,β-unsaturated aldehyde (46, 48). The result showed that UIP migrated differently from the products defined by the enzymes EcNth, EcFpg, and hAPE1, but identical to the product formed by hOGG1 (**Figure 1H**), suggesting that the UNG-generated product is a 3´-α,β-unsaturated aldehyde. The product corresponds to that obtained with hSMUG1 (38), which we decided to designate uracil-DNA incision product (UIP).

### Direct Identification of the hUNG Incision Products as a 3´-α,β-Unsaturated Aldehyde and a 5´-Phosphate

**Figure 2:**
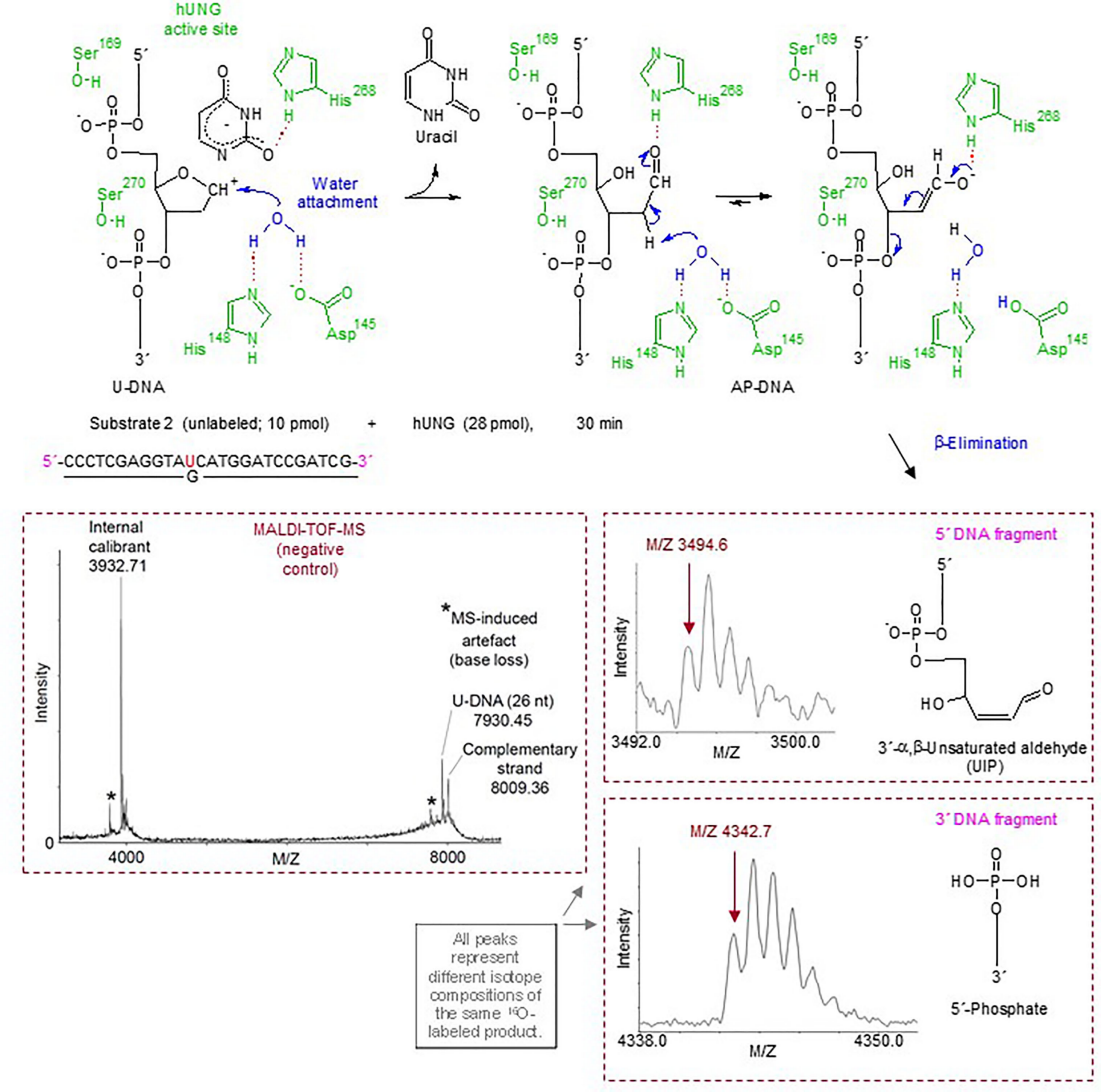
Suggested hUNG reaction mechanism for incision of U-DNA into UIP and 5´-phosphate as identified by MALDI-TOF-MS and supported by structural modeling and site-directed mutagenesis. In the upper panel, amino acid residues of hUNG involved in catalysis are indicated in green; their hydrogen bonds with catalytic water and substrate are indicated by red dotted lines. Enzymatically activated H_2_O (in blue) is attached at C1´ of the abasic deoxyribose [blue arrow; based on refs. (49, 50)]. Proposed electronic (that cause atomic) transfers that are involved in the formation of UIP are indicated by short blue arrows. A hydrogen bond between His268 and the formyl oxygen of the ring-opened abasic deoxyribose (51) is proposed. We suggest that Asp145 acts as a general base, and that the attraction of electrons by the C1´ formyl group improves the C2´ hydrogen as a leaving group. The middle and lower right panels show the MALDI-TOF-MS signals of the 5´ and 3´ DNA incision fragments, respectively, while the middle left panel shows the signals obtained from incubation without enzyme.

The quantitative but indirect method of gel electrophoresis was supplied by MALDI-TOF-MS analysis for direct chemical identification of the BER cleavage products (38), employing an unlabeled version of substrate 2 exposed to hUNG (**Figure 2**, upper left panel) as well as the enzymes used to define the different 3´-end products [see **Figure 1H**; apart from that *E. coli* endonuclease IV (EcNfo) was used to define the 3´-OH product instead of hAPE1]. We included incubations in solutions made in H_2_^18^O to indicate reaction mechanism. The results showed that hUNG produced a 5´-DNA fragment of M/Z 3494.6, corresponding to the mass of a fragment containing a 3´-α,β-unsaturated aldehyde (**Figure 2**, middle right panel). This was also the case for hOGG1 and hSMUG1, as previously reported (38), as opposed to EcFpg and EcNfo, which did not form such a DNA fragment (**Supplementary Figure 2**). These results demonstrate that the hUNG-mediated U-DNA incision activity cannot be explained by AP endonuclease contamination of the purified enzyme preparation. Importantly, a signal of M/Z 3512.6 also appeared following hUNG digestion, even though enzyme reactions were carried out in H_2_^18^O (**Supplementary Figure 3**, left). This can be explained by post-enzymatic addition of water (mostly H_2_^16^O) to the 3´-α,β-unsaturated aldehyde, since such addition during enzyme reaction (mostly H_2_^18^O) should result in a product of M/Z 3514.6 (due to a 3´-^18^OH group). However, when the enzymatically exposed substrate was precipitated with ethanol in the presence of ammonium acetate instead of sodium acetate, a signal corresponding to M/Z 3511.6 appeared while the “M/Z 3512.6” product was absent. This can be explained by quantitative addition of ammonia to the double bond of the 3´-α,β-unsaturated aldehyde (**Supplementary Figure 3**, middle). When the reaction products were dissolved in H_2_^18^O instead of normal water, the M/Z 3511.6 signal decreased in favor of a signal corresponding to M/Z 3513.6. This accords with the presence of an aldehyde group, which exchanges oxygen isotopes by addition–elimination of water, at C1´ (**Supplementary Figure 3**, right). Thus, in addition to directly identifying a fragment with the same molecular weight as if it contains a 3´-α,β-unsaturated aldehyde (**Figure 2**, middle right panel), our results demonstrate two possible post-enzymatic derivatives of this product (**Supplementary Figure 3**). This confirms the presence of a double bond and provides compelling evidence that the 5´ incision fragment formed by hUNG is indeed a 3´-α,β-unsaturated aldehyde. The MALDI-TOF-MS analysis also showed that incubations with hUNG, like those with EcFpg, produced a signal corresponding to M/Z 3396.6 (**Supplementary Figure 2**), which corresponds to the mass of a 5´-DNA fragment with a 3´-phosphate. Thus, it seems like hUNG, as hSMUG1 (38), is able to form a 3´-phosphate end, although at a very low concentration since it is hardly detectable in the quantitative PAGE experiments (**Figure 1H**). Alternatively, some 3´-α,β-unsaturated aldehyde may be converted chemically to 3´-phosphate during sample preparation. Importantly, we observed a signal of M/Z 4342.7 in all experiments, regardless whether or when we used ^18^O- or ^16^O-water or ammonium-based precipitation. This M/Z value corresponds to a 3´-fragment containing a 5´-phosphate end (**Figure 2**, lower right panel). No ^18^O-incorporation took place at the 5´-phosphate. As previously described for hSMUG1, we did not observe any signal corresponding to a 5´-fragment containing a 3´-dUMP, which indicates that the formation of UIP follows uracil excision. We also did not observe any signal corresponding to the masses of UIP or other possible U-DNA incision or processing products in control incubation without repair enzyme (**Figure 2**, lower left panel). Finally, we observed a signal of M/Z 3316.5 corresponding to a 3´-OH when substrate subjected to hUNG was further incubated with hAPE1 (**Supplementary Figure 4**), as previously demonstrated by PAGE (**Figure 1H**).

### Incision Products and Active Site Structure Suggest a β-Elimination Mechanism Involving the Same Amino Acid Residues That Participate in Uracil Excision

Enzyme reactions performed in the presence of H_2_^16^O and H_2_^18^O (**Supplementary Figure 3**) were consistent with a β-elimination reaction mechanism (**Figure 2**, upper middle and right panels). However, the failure to trap a UDG–DNA reaction intermediate as a stable covalent complex (**Supplementary Figure 5**) and the fact that hUNG lacks an active site lysine (52–54) to carry out a β- or a β/δ-elimination reaction indicate that the excision and incision activities are not concerted.

**Figure 3:**
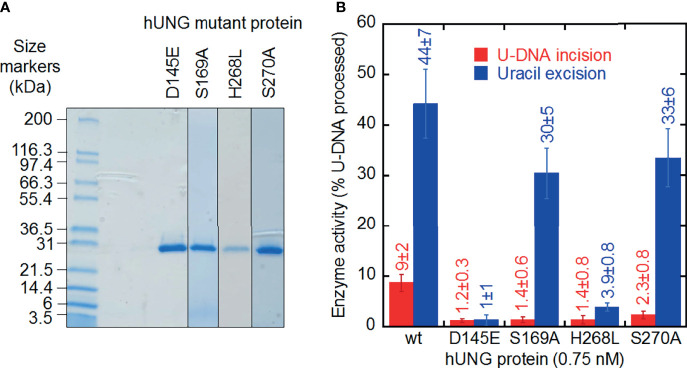
U-DNA incision and uracil excision activity of hUNG mutant proteins purified to apparent physical homogeneity. **(A)** SDS-PAGE of purified proteins stained with Coomassie Blue. Samples (20 µl; treated 5 min at 95°C in NuPAGE^®^ LDS Sample Buffer, Cat. No. NP0007, Life Technologies) and protein markers (Mark12™ Unstained Standard, Cat. No. LC5677, Life Technologies) were run on a 4–20% Mini-PROTEAN^®^ TGX™ Precast Gel (12 wells; Bio-Rad) in 25 mM Tris, 0.192 M glycine, 0.1% (w/v) SDS at 200 V for 35 min. Left lane, protein markers; right lanes, hUNG mutant proteins. **(B)** Different decrease in U-DNA incision and uracil excision activity by site-directed mutant hUNG proteins. Wild-type and mutant proteins (0.015 pmol) were incubated with S1 (10 pmol) at 37°C for 20 min (see **Figure 1A**). Each value represents the average ± SD of 5–16 independent measurements.

**Figure 4:**
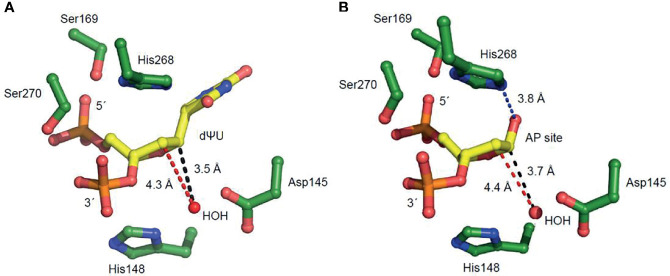
Amino acid residues of hUNG active site region positioned to participate in uracil excision and uracil-DNA incision. These enlarged views in which 2´-deoxyuridine is replaced with **(A)** 2´-deoxypseudouridine (dΨU) or **(B)** an AP site indicate amino acid residues involved in substrate binding and coordination. The distances between the catalytic water molecule and the site C1´ where it is attached (causing uracil excision) and the site C2´ of proton elimination (causing uracil-DNA incision) are indicated by black and red broken lines, respectively. The His268 NH–C1´O distance in **(B)**, facilitating proton removal from C2´, is indicated by a blue broken line.

A crucial question is whether the U-DNA incision activity of hUNG is dependent on the same or different amino acid residues than the uracil excision activity. Thus, a number of hUNG site-directed mutant proteins were overproduced in *E. coli ung*^‒^, purified (41) to apparent physical homogeneity (**Figure 3A**) followed by confirmation of correct amino acid replacements by LC-MS analysis (**Supplementary Figure 6**), and subsequently biochemically characterized with respect to the two activities using the same substrate (**Figure 3B**). The amino acid residues selected for replacement were chosen based on analysis of the crystal structure of hUNG in complex with dsDNA containing the non-hydrolyzable substrate analog 2´-deoxypseudouridine (dΨU) (PDB1emh) (43), as well as the hUNG mutant protein Leu272Arg/Asp145Asn in complex with dsDNA where the uracil–deoxyribose bond was cleaved (44). All residues mutated are either directly involved in catalysis, coordinating the flipped-out uracil nucleoside or known to coordinate phosphate groups in DNA. They are all evolutionally conserved among the human, mouse (8), *Caenorhabditis elegans* (55), *Saccharomyces cerevisiae* (56), and *E. coli* (57) UNG enzymes (**Supplementary Figure 7**). In the best mimic to the U-DNA/hUNG structure before the excision of uracil, structural analysis shows lengths of 3.5 Å and 4.3 Å between the catalytic water molecule and the deoxyribose C1´ and C2´ of the flipped-out (un-excisable) dΨU (43) (**Figure 4A**), respectively, which are quite similar distances for reaction. In the best mimic to the AP-DNA/hUNG structure before the formation of UIP, the analogous lengths are 3.7 Å and 4.4 Å (44) (**Figure 4B**), i.e., nearly identical to the former. We also believe that the proposed β-elimination reaction taking place after glycosidic bond cleavage is enabled by more space and flexibility for atomic transfers than the uracil excision reaction, allowing entry of another catalytic water molecule. Thus, in a crystal of the mutant Leu272Ala hUNG protein and AP-DNA the uracil-binding pocket was filled with two water molecules (44), indicating increased water accessibility following release of the free base from the active site. Half of the hUNG mutants show significant loss of activity with respect to U-DNA incision and uracil excision (**Figure 3B**) arguing that the two reactions are coupled. The dramatic decrease in both activities by replacement of Asp145, which has been suggested to activate water for attack at C1´ to release uracil (**Figure 2**, upper left panel) (40), accords with our suggestion that this residue is also important for activating a water molecule to abstract a proton from C2´ resulting in strand incision (**Figure 2**, upper middle panel). Interestingly, the replacement of His268 causes a similar effect (**Figure 3B**), and we want to propose that His268 facilitates proton removal from C2´ by coordinating the formyl oxygen in the ring-opened form of the abasic deoxyribose (58), enhancing the electron-withdrawing effect of the formyl group (**Figure 2**, upper middle panel). The importance of His268 for both uracil excision and U-DNA incision can thus be explained by a similar, although not identical function in catalysis, which is coordination of a carbonyl oxygen (**Figure 2**, upper panel). This accords with a distance of 3.8 Å between the His268 NH and the deoxyribose C1´ oxygen in the best mimic to the AP-DNA/hUNG structure before the formation of UIP (**Figure 4B**). The importance of Asp145 and His268 in the elimination reaction presented here accords with common roles established for these residues in facilitating hydrolysis and dehydration reactions through proton shuttling and electrostatic stabilization (59).

### Ser169/Ser270 Phosphate Interactions Seem Crucial for U-DNA Incision as Opposed to Uracil Excision

The Ser169Ala and Ser270Ala proteins represent interesting exceptions to the observed trend of a similar decrease in uracil excision and U-DNA incision activity caused by an amino acid replacement. In these two cases, the incision activity is considerably affected while the excision activity is comparable to wild-type hUNG (**Figure 3B**). It has been proposed that their effects on uracil excision are due to interactions of Ser169 with the 5´-phosphate and Ser270 with the 3´-phosphate (**Figure 4**) promoting the “flipping out” movement (16). Principally, this should also affect the incision reaction since the substrate for this is the product of the excision reaction. Indeed, after the excision reaction has occurred, such a Ser–phosphate interaction seems to be even more important for the structural adjustments that need to take place before the C2´ atom of the abasic deoxyribose and a water molecule are correctly oriented to react. This possibly explains the large decrease in U-DNA incision activity when one of these serine residues has been inactivated.

## Discussion

**Figure 5:**
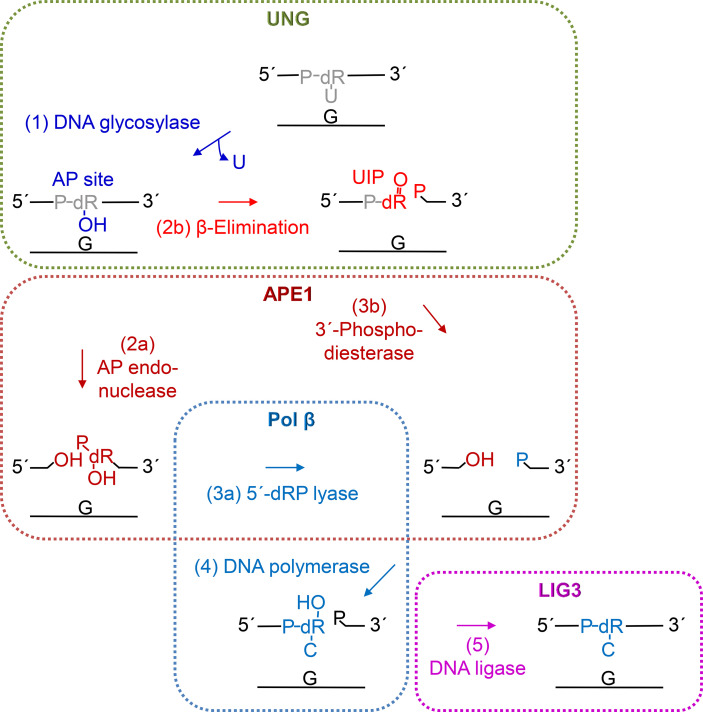
Proposed steps of hUNG-initiated uracil BER. After uracil is removed (step 1, blue) by the DNA glycosylase activity of UNG (green), the AP site is mostly incised (step 2a) by APE1 (dark red) leaving behind a 3´-OH group. Then, the 5´-deoxyribose (dR) phosphate (P) remnant is removed by the dRP lyase activity (step 3a) of DNA polymerase β (Pol β, light blue), following conclusion of BER by insertion of the correct dCMP (step 4) by Pol β and nick-sealing (step 5) by DNA ligase III (LIG3, purple). Alternatively, UNG may itself incise the AP site (step 2b, red) by β-elimination (**Figure 2**, upper middle and right panels) leaving behind a 3´-α,β-unsaturated aldehyde (UIP; **Figure 2**, middle right panel), which can be removed by the 3´-phosphodiesterase activity (step 3b) of APE1, and a 5´-P (**Figure 2**, lower right panel).

In this report we demonstrate that hUNG—hitherto regarded as a mono-functional DNA glycosylase—is able to incise the phosphodiester backbone of DNA at the uracil site after uracil excision (**Figure 1B**). The activity is coupled to uracil excision, since no incision was detected on DNA with uracil exchanged by an AP site (**Figure 1G**). We recently reported the same for hSMUG1, which encouraged us to call it UIP (38). The UIP formed by hUNG is processed to form 3´-OH product by hAPE1 *in vitro* (**Supplementary Figure 4**). This suggests efficient downstream processing *in vivo* by the BER pathway (**Figure 5**). Our results on hSMUG1 and now hUNG add to other recent findings, as, e.g., that human poly(ADP-ribose) polymerase-1 efficiently binds AP sites and also exhibits AP lyase activity (60) and that hAPE1 has a high affinity for and is able to incise—although at an extremely low rate—U-DNA, leaving behind a 5′-terminal dUMP residue (61).

U-DNA incision by hUNG is best explained by a β-elimination reaction at the C2´–C3´ bond, albeit without the formation of an imine intermediate as indicated by the sodium borohydride trapping experiment performed according to Zharkov et al. (62) (**Supplementary Figure 5**) (38). Theoretically, the elimination reaction may occur *via* deprotonation of C2´ leading to formation of the enolate intermediate, although the O1´ negative charge may require stabilization. This can, indeed, explain UIP formation in one step, probably involving the same hUNG amino acid residues that are needed by the uracil excision reaction (**Figure 2**, upper panel) (49, 50). One of the original models for catalysis of base excision by family 1 UDGs suggested an associative SN2 mechanism, which shortly says that following flipping into the active site uracil is released from deoxyribose by attack on the C1´ of a water molecule activated by Asp145 in hUNG acting as a general base (with possible assistance from His148) (**Figure 4A**) (16, 40, 63). In contrast, later results supported by biophysical investigations have favored a dissociative SN1-like mechanism, which means that following base flipping into the active site, the glycosidic bond splits into a uracil anion stabilized by a histidine residue and a deoxyribose oxocarbenium ion. Then, a water molecule, coordinated by certain active site amino acid residues, somewhat passively becomes the 1´-α-OH C1´ after dissociation of the uracil anion (49) (**Figure 2**, upper left panel). While the SN1 approach focuses on the activation of a H_2_O nucleophile by certain amino acid residues (16), the SN2 model emphasizes the reaction energy contributed by molecular strain or other unfavorable atomic clashes in U-DNA before and following base flipping (43). In our tentative reaction mechanism, we employ the SN1 model for uracil excision at the same time as we propose a role for Asp145 in activating a water molecule to deprotonate C2´, which also relates to the SN2 approach (**Figure 2**, upper panel).

To substantiate this hypothesis, we produced a number of site-directed mutant proteins (**Figure 3A**) and assayed them for uracil excision and U-DNA incision activity (**Figure 3B**). This was based on inspection of hUNG crystal structures complexed with substrate analogs or an AP site (43). It is important to note that the lengths between a (proposed) catalytic water molecule and the deoxyribose C1´ and C2´ are virtually identical in these structures. These lengths were 3.5 Å and 4.3 Å before uracil excision (**Figure 4A**), and 3.7 Å and 4.4 Å after (**Figure 4B**), respectively. The uracil excision and U-DNA incision reactions are coupled (**Figure 2**, upper panel). It is consequently difficult to inactivate the former without affecting the latter. The Asp145Glu or His268Leu mutant proteins therefore cause a dramatic decrease in both activities (**Figure 3B**). The water molecule, Asp145/His148, and C2´ seem too far apart for deprotonation in the DNA incision reaction. The ability of Asp145 to move from an open to a closed position (43), and the existence of deoxyribose in a ring-closed and an open structure (51) therefore suggests a flexibility that may make the C2´ deprotonation feasible. His268 also seems to be close enough to stabilize the enolate intermediate (**Figure 2**, upper right panel). The proposed β-elimination therefore implies less spatial constraints, higher atomic transfer flexibility and increased (catalytic) water accessibility than the uracil excision reaction. This is supported by the crystal structure of the Leu272Ala mutant protein complexed with AP-DNA. The active site pocket is filled with two instead of one water molecule, and attachment to the AP site compresses, like ordinary unspecific DNA binding, the DNA backbone to promote molecular displacements like nucleotide flipping (44). It is suggested that interactions between Ser169 and the 5´-phosphate, and Ser270 and the 3´-phosphate (**Figure 2**, upper left panel) facilitate uracil excision by promoting the “flipping out” movement (16). Our results suggest that such Ser–phosphate interactions might also be important to the structural adjustments needed for the β-elimination reaction, since the Ser169Ala and Ser270Ala mutant proteins show a much larger decrease in incision than in excision activity, the latter being comparable to that of wild-type hUNG (**Figure 3B**). In crystals of hUNG protein in complex with product AP-DNA and free uracil as well as mutant Leu272Ala protein and AP-DNA, Parikh et al. did not observe cleaved DNA (44). Although comparison of results between so different methods (crystallography versus biochemical assay) is challenging, different experimental conditions like different opposite bases to uracil (they used A instead of G) and incubation buffers may cause discrepancies. It is also possible that the protein–DNA complex dissociates after backbone incision, or that, even if it is stable, it is sufficiently different not to crystallize under the conditions used.

The mechanism presented (**Figure 2**, upper panel) is new and therefore interesting in itself, but also interesting because it opens a possibility of other reactions that uracil BER can engage in (**Figure 5**). However, we believe that even more exciting is the implications for DNA nick generation in immunoglobulin gene diversification. The relative importance of the two AP endonucleases, APE1 and APE2, in CSR is still uncertain. It has been suggested that low levels of APE1 in germinal center cells (64) indicate that APE2 might be the primary nicking enzyme, even though APE2 exhibits much lower AP endonuclease activity than APE1 (65, 66). The essential nature of *Ape1* has made genetic experiments in animals difficult (67). Masani et al. (68) instead constructed B cells deficient in Ape1, Ape2, and both. In this system, CSR efficiency was similar in wild-type and Ape2-deficient cells but reduced to ~20% of wild-type levels in the Ape1-deficient cells. No additive effect was found when the *Ape2* gene was inactivated, suggesting that CSR requires Ape1. The substantial (~20%) residual CSR in the absence of both AP endonucleases suggests that other enzymes may be contributing DNA nicking activity. Consistently, AID-induced DSB levels were not reduced in *Ape1* null B-cells (68) suggesting that Ape1 is dispensable for the generation of AID-dependent DNA breaks in the switch (S) region but might be required for the subsequent end processing (69). The results of Zarrin et al. (70) show that CSR can be supported by even a single DSB in Sμ and a downstream S region. This leads us to question the absolute requirement of the high AP nicking efficiency provided by APE1. It can further be argued, based on this, that nick generation may take place less systematically than we currently believe. Consequently, it remains unclear which enzymes provide strand cleavage for the further processing of AID and UNG-generated AP sites.

**Figure 6:**
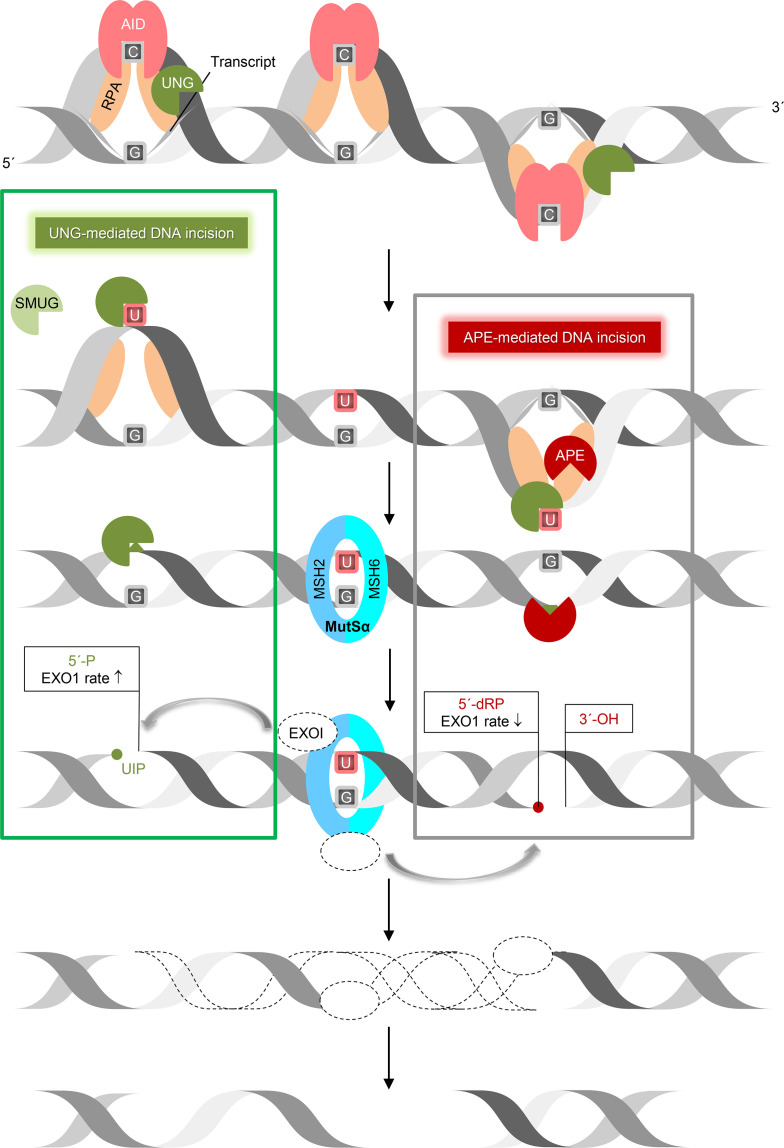
UNG-mediated DNA incision in CSR. This working model suggests how removal of AID-generated uracil followed by incision of the AP site by UNG and nick processing by exonuclease 1 (EXO1) form DSB in immunoglobulin switch regions. Transcription of the targeted immunoglobulin gene region forms bubbles in DNA, so granting AID access to ssDNA (stabilized by RPA) to deaminate C to U. This results in UNG recruitment (by RPA) and uracil excision. According to our results (left square), UNG (with SMUG1 as a backup) is able to incise the AP site, leaving behind a 5´-phosphate, which is a better substrate for exonuclease 1 (EXO1) 5´→ 3´ digestion than the 5´-deoxyribose phosphate left behind by APE1 incision (right square). This model relies on the MMR component MutSα (MSH2/6), which recognizes a U:G mismatch and recruits EXO1. This also applies to ssDNA patch generation by EXO1 in SHM. ↑, increased, ↓, decreased.

We propose, based on the inconclusive data on the precise role of APE1 in CSR, and on our demonstration of U-DNA nicking activity of hUNG (**Figure 1**), that UNG might contribute to AP site incision, thus providing the initial nicked intermediate required for CSR. The observation that mutated *UNG* genes in B cells from patients with the hyper-IgM syndrome produce proteins deficient in uracil excision from ssDNA but not dsDNA (37) may also relate to our finding that the incision activity of hUNG fully applies to uracil in ssDNA (**Figure 1E**)—the direct product of AID in CSR (and SHM). It is therefore possible that UNG may compete with APE1 in the carrying out of the AP site incision function while APE1, as mentioned above, provides further strand processing activity (65, 66). This accords well with the results of the Kobayashi/Honjo group (69). It also explains remaining DNA nicking activity in CSR of murine cells following the deletion of all Ape1 and Ape2 activity (68). Another possibility is that nicks generated by UNG may funnel substrates towards error-prone processing. The 5´-phosphate left behind by UNG (**Figure 2**, lower right panel) is a preferred substrate for 5´→ 3´ exonuclease 1 (EXO1) rather than the 5´-dRP left behind by APE1 (71). UNG-mediated nick generation might therefore suppress error-free BER at productive S loci, possibly resulting in more efficient generation of DSB than APE1 incision (**Figure 6**). This model would be consistent with collaboration between UNG and the mismatch repair (MMR) system consisting of MutSα, MutLα (MLH1 and PMS2), and EXO1 (72), for nick and ssDNA generation in CSR and SHM (33, 73). Our results are therefore not in conflict with the long-patch BER or the hybrid BER/MMR models. However, the results presented here (**Figures 1**–**3**), and previous results (38), question the absolute need of involving AP endonucleases in CSR. The ability of UNG to nick DNA may also partially explain the reported dispensability of APE enzymes in SHM through providing MMR complex accessibility at an earlier stage. There might, therefore, be a need to reinterpret experiments on B cells deficient in UNG, and to consider the ability of different relevant enzymes to generate nicks in DNA processed by AID. Further investigation on how UNG and other UDGs (30–32, 38, 74) participate in CSR and SHM should include *in vitro* experiments both with and without the presence of AID, APE1, and/or APE2. These experiments should use DNA substrates that mimic the S and variable immunoglobulin gene regions. Genetic experiments with plasmids that express differently mutated *UNG* genes in *Ung^−/−^* B cells should also be conducted.

In summary, the seminal discovery of AID and its crucial importance in immunoglobulin gene formation have, in the last two decades, largely determined the agenda for studies in molecular immunology (25). Our results are compatible with the classical model in which UNG prepares AID-induced uracil for incision by excising uracil (**Figure 6**, right). Our data, however, suggests that UNG also provides strand incision activity (**Figure 6**, left).

## Data Availability Statement

The datasets presented in this study can be found in online repositories. The names of the repository/repositories and accession number(s) can be found in the article/**Supplementary Material**.

## Author Contributions

MA and MNM designed and performed experiments, analyzed the data, and revised the manuscript. XMX designed and performed experiments and analyzed the data. AR performed the MS experiments and analyzed the data. IL performed computer-assisted molecular modeling and wrote the manuscript. FK planned the MS experiments and analyzed the data. LA and HN analyzed the data and wrote the manuscript. AK designed the experiments, analyzed the data, supervised the study, and wrote the manuscript. SB designed the experiments, analyzed the data, supervised and managed the study, and wrote the manuscript. All authors contributed to the article and approved the submitted version.

## Funding

This research was supported by the University of Stavanger, Oslo University Hospital/University of Oslo, and Akershus University Hospital.

## Conflict of Interest

The authors declare that the research was conducted in the absence of any commercial or financial relationships that could be construed as a potential conflict of interest.

## Publisher’s Note

All claims expressed in this article are solely those of the authors and do not necessarily represent those of their affiliated organizations, or those of the publisher, the editors and the reviewers. Any product that may be evaluated in this article, or claim that may be made by its manufacturer, is not guaranteed or endorsed by the publisher.
