# Supplementary material for: Intrinsic Strand-Incision Activity of Human UNG: Implications for Nick Generation in Immunoglobulin Gene Diversification
Source: Front Immunol. 2021 Dec 22;12:762032. doi: 10.3389/fimmu.2021.762032 (PMC8730318; doi:10.3389/fimmu.2021.762032)
Supplement: Supplementary file 1 [file Presentation_1.pdf]

Supplementary Material

Intrinsic Strand-Incision Activity of Human UNG Predicts Nick Generation in Immunoglobulin Gene Diversification

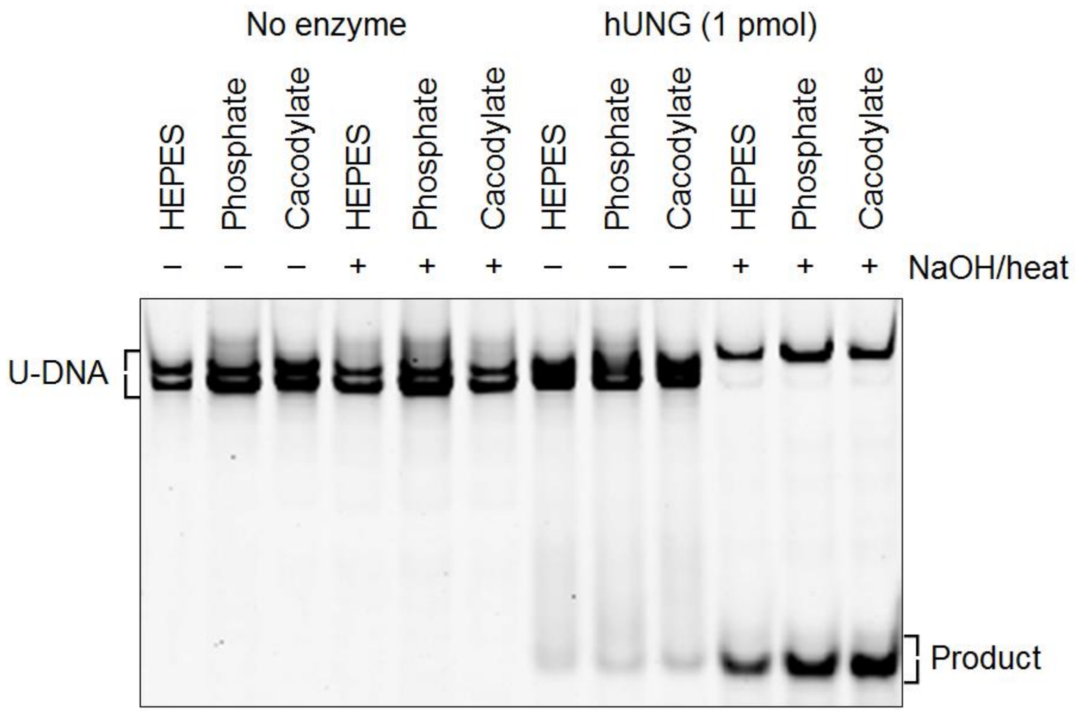

Supplementary Figure 1 | U-DNA incision by hUNG in different buffers. U-DNA (1 pmol of substrate 1) was incubated with hUNG (1 pmol; upper panel, last 6 lanes) or without enzyme (upper panel, first 6 lanes) in reaction buffer (HEPES), or in 45 mM sodium cacodylate, or 45 mM potassium phosphate buffer, with the same pH and additions as for reaction buffer (see Materials and Methods), at 37°C for 10 min (final volume, 20 µl). Samples were prepared as described in Materials and Methods. Incision product was separated from un-incised DNA by PAGE at 120 V for 2 h using a 20% (w/v) gel with 3% (v/v) formamide. The picture shows a typical experiment (5 experiments performed; see **Figure 1F** for graph of the results).

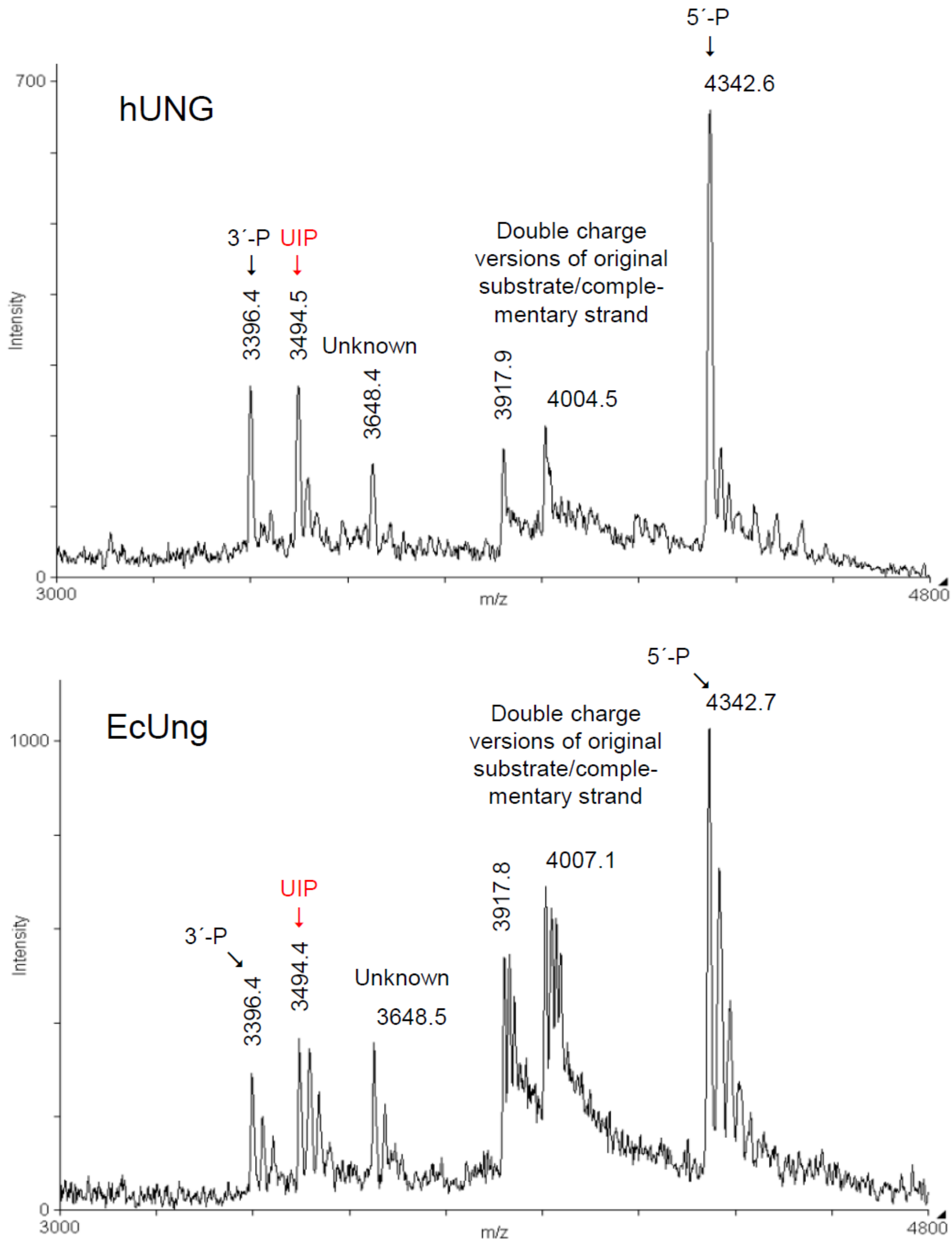

hUNG
700
Intensity
0
3000
m/z
4800
5´-P
4342.6
3´-P
UIP
3396.4
3494.5
Unknown
3648.4
Double charge versions of original substrate/comple-mentary strand
3917.9
4004.5
EcUng
1000
Intensity
0
3000
m/z
4800
5´-P
4342.7
Double charge versions of original substrate/comple-mentary strand
4007.1
3917.8
UIP
3´-P
3494.4
3396.4
Unknown
3648.5

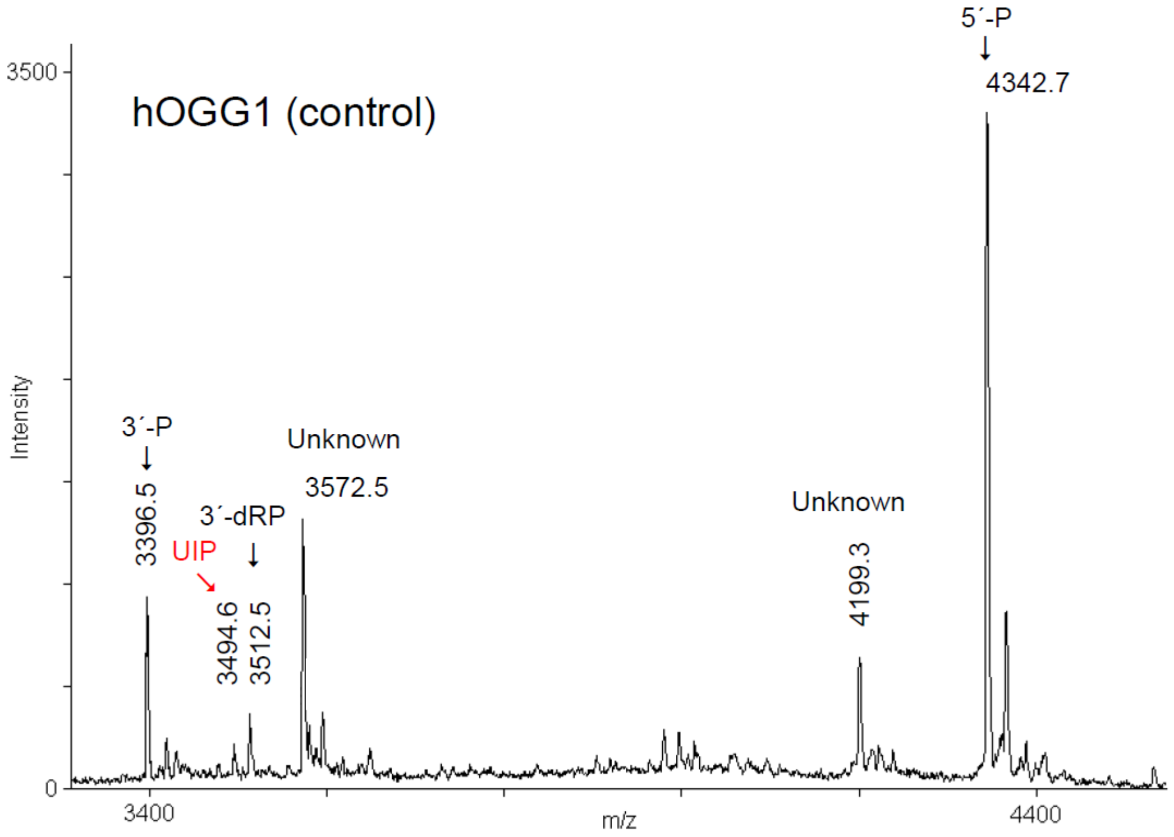
hOGG1 (control)
3500
Intensity
0
3´-P
3396.5
UIP
3494.6
3´-dRP
3512.5
Unknown
3572.5
Unknown
4199.3
5´-P
4342.7
3400
4400
m/z

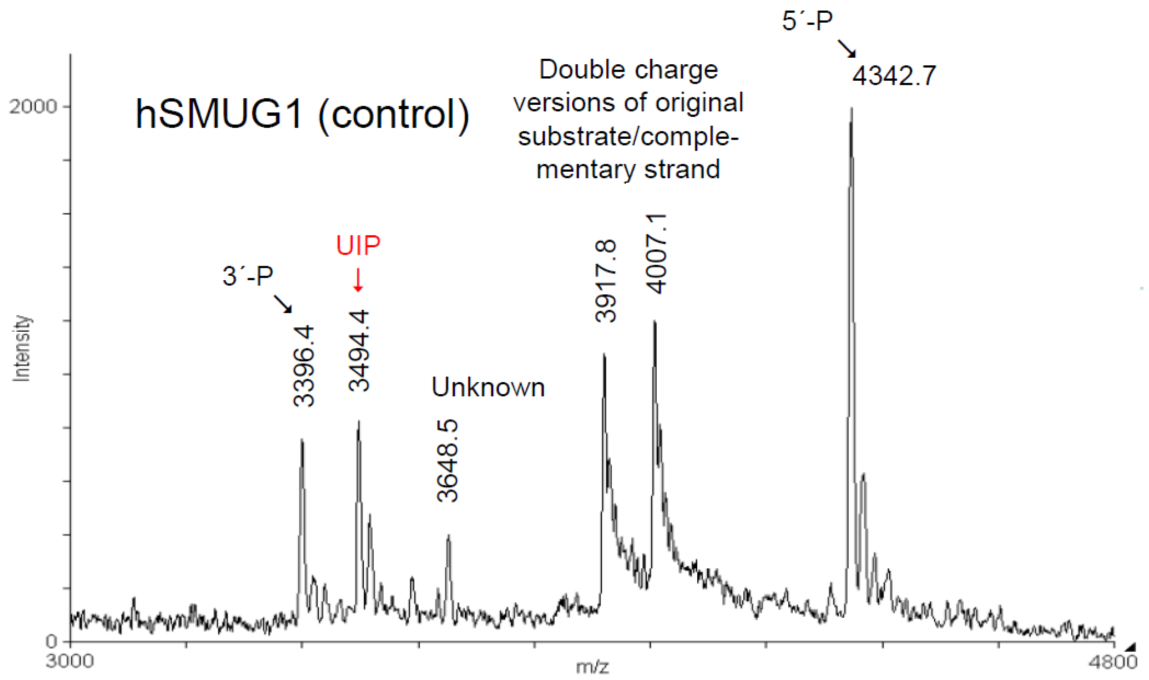
hSMUG1 (control)
2000
Intensity
0
3´-P
3396.4
UIP
3494.4
Unknown
3648.5
Double charge versions of original substrate/complementary strand
3917.8
4007.1
5´-P
4342.7
3000
4800
m/z

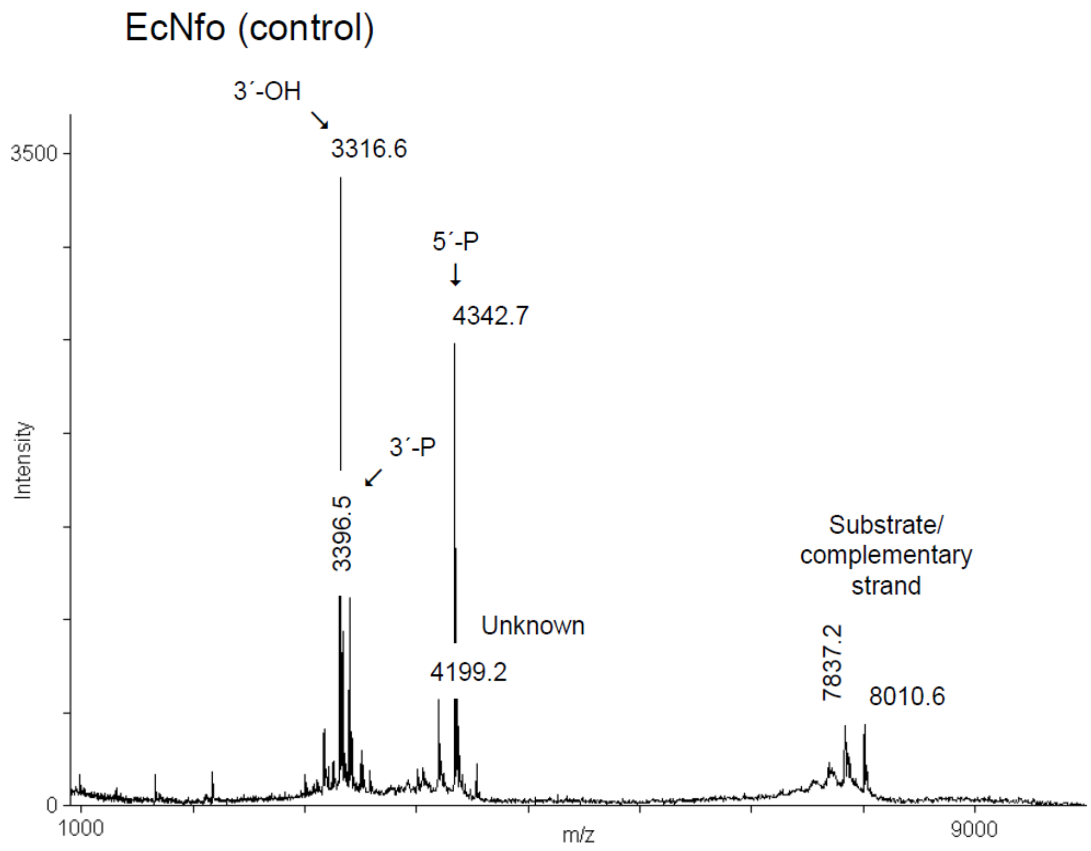
EcNfo (control)
3´-OH
3316.6
5´-P
4342.7
3´-P
3396.5
Intensity
3500
0
Substrate/
complementary
strand
Unknown
4199.2
7837.2
8010.6
1000
m/z
9000

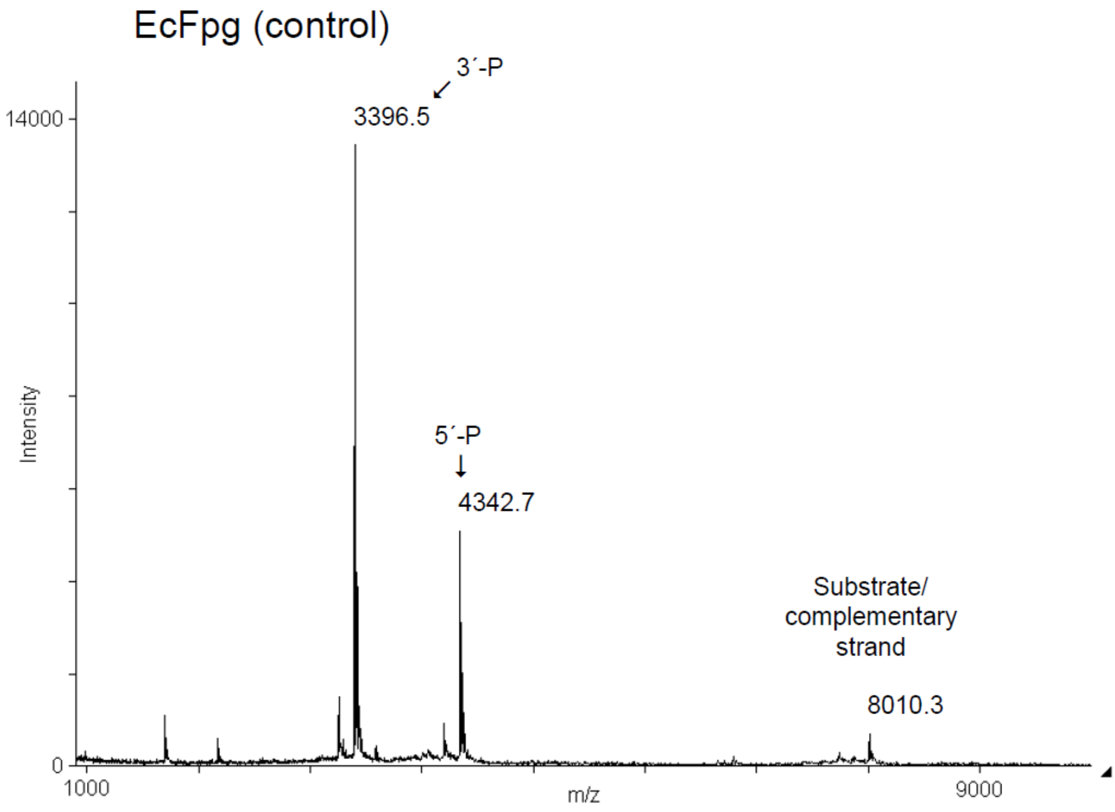
EcFpg (control)
3´-P
3396.5
14000
Intensity
5´-P
4342.7
Substrate/
complementary
strand
8010.3
0
1000
m/z
9000

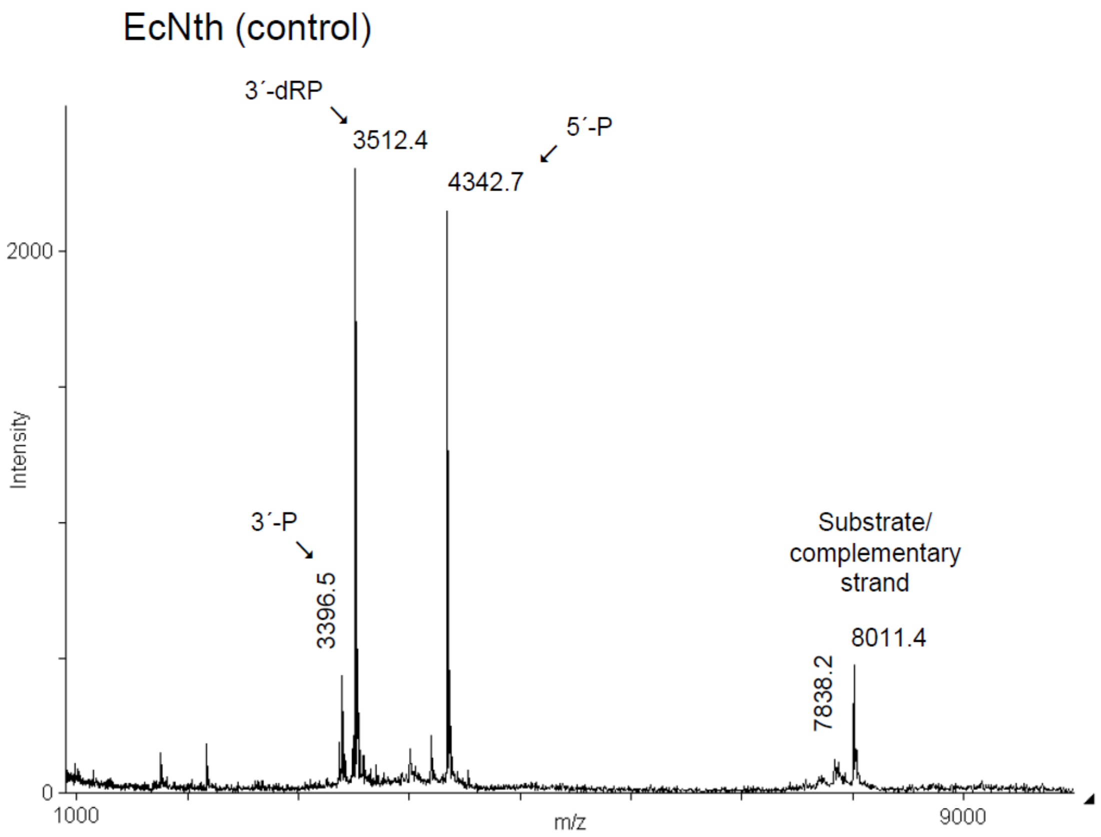

Supplementary Figure 2 | Confirmation of the chemical nature of UIP by MALDI-TOF-MS after hUNG treatment of U-DNA. UIP was mostly detected as the 3´-α,β-unsaturated aldehyde itself (M/Z 3494.6) by hUNG and EcUng; however, the aldehyde can also be converted to its hydrated derivative by post-enzymatic addition of H_2O (3´-dRP; M/Z 3512.6; see **Supplementary Figure 3**). hOGG1 and EcNth (used as positive controls) both cause 3´- α,β-unsaturated aldehyde and 3´-dRP (Ref. 38), where varying amounts of them were detected by PAGE (**Figure 1H**). EcNfo (used as control) mostly causes 3´-OH. 3´-P (M/Z 3396.6) was detected after exposure to all enzymes: In the case of hUNG and EcUng it might be formed by long-time exposure, which was the case for hSMUG1 (Ref. 38). However, because it is not significantly detected by the short-time exposure employed (**Figures 1B, H**), and is probably of limited impact *in vivo*, the possible enzymatic formation of 3´-P by hUNG was not further investigated. 3´-P may also be formed chemically/physically during MS, e.g. by fragmentation of UIP. All experiments were performed in the presence of normal H_2O; DNA was precipitated in the presence of sodium acetate (Ref. 38).

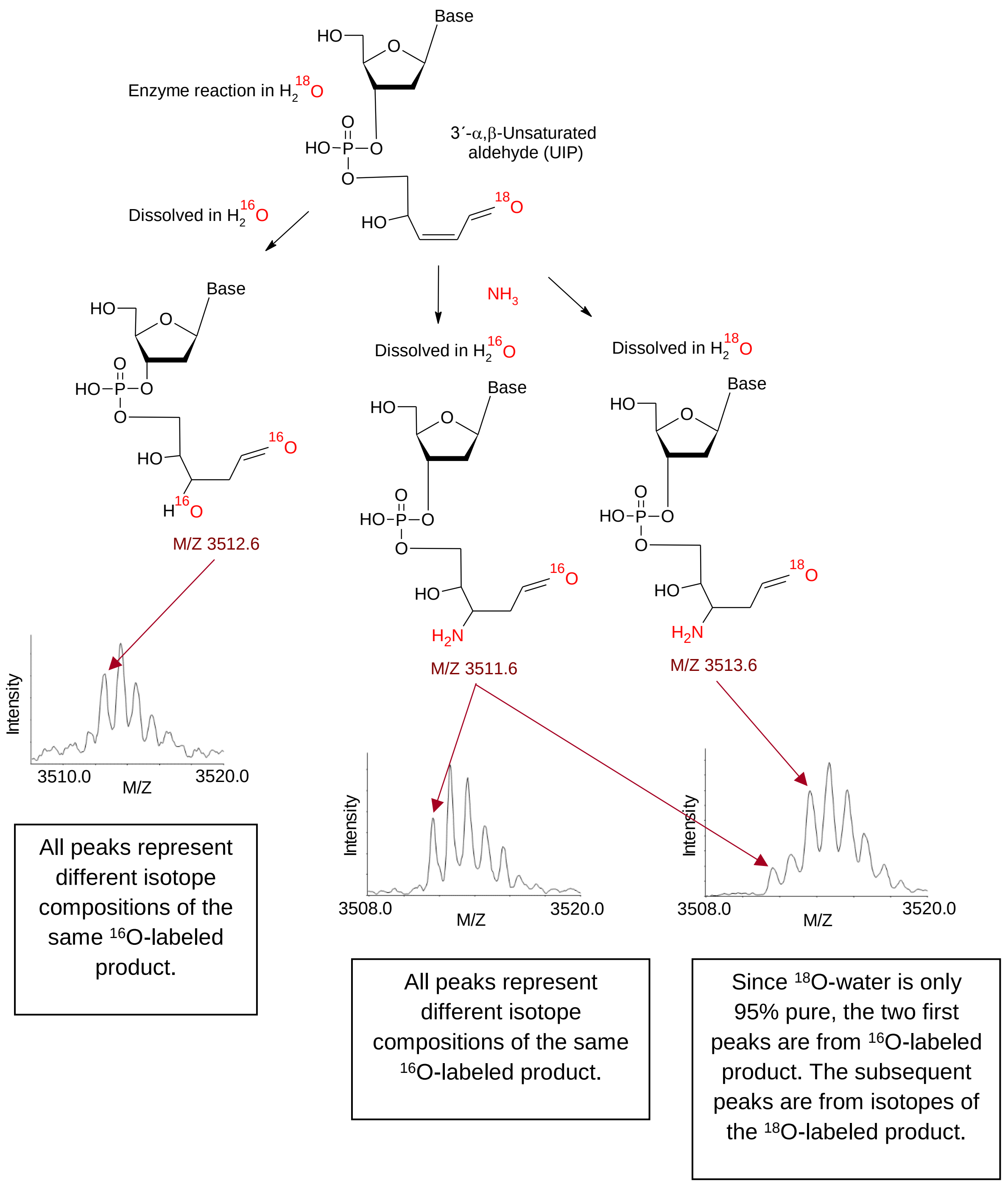

Supplementary Figure 3 | Confirmation of the chemical nature of UIP derivatives by MALDI-TOF-MS after hUNG treatment of U-DNA in ^{18}O water ($H_2^{18}O$). The observed post-enzymatic addition of ^{16}O-water (left) or ammonia (middle and right) can be explained by reaction with the conjugated double bond, while the efficient exchange of an oxygen atom when the sample was transferred from ^{18}O- to ^{16}O-water (left and middle) can be explained by the presence of an aldehyde group (Ref. 38). The MALDI-TOF-MS (monoisotopic; dark red arrows) signals of the different chemical structures are shown in the lower panel

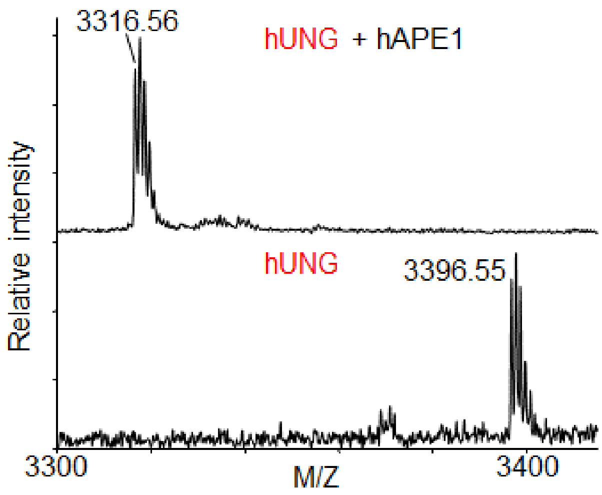

Supplementary Figure 4 | MALDI-TOF-MS signals for incubation of U-DNA with hUNG with and without hAPE1.

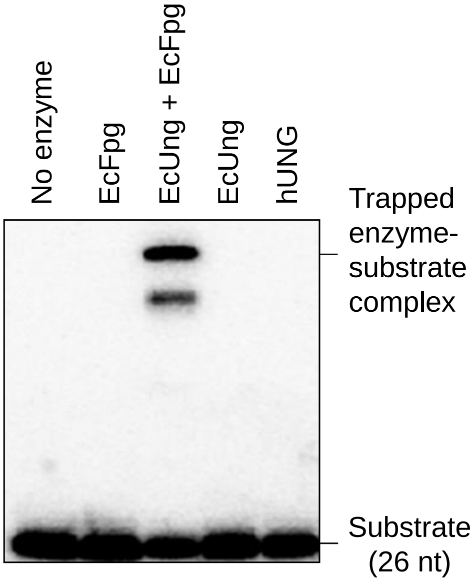

Supplementary Figure 5 | Trapping experiment for Schiff base intermediate (Ref. 62). Enzyme (EcFpg, 17 pmol; EcUng, 3 pmol; hUNG, 5 pmol) was incubated with substrate 2 (1 pmol) and freshly dissolved 50 mM $NaBH_4$ in reaction buffer at 37°C for 10 min (final volume, 10 µl). Reaction was terminated by the addition of 10 µl loading solution (NuPAGE® LDS Sample buffer containing NuPAGE® Reducing agent, Invitrogen, 1× final) and heated at 70°C for 10 min before loading (15 µl) onto a SDS-PAGE [10% (w/v)] gel (NuPAGE® Novex® Bis-Tris Mini gel, Invitrogen). Electrophoresis was performed at 200 V for 50 min in Mops buffer (1×) (NuPAGE® MOPS SDS Running Buffer 20×, NP0001, Invitrogen). Visualization was performed by phosphorimaging analysis as before. A similar experiment was also performed using 1 h incubation time showing the same result (data not shown).

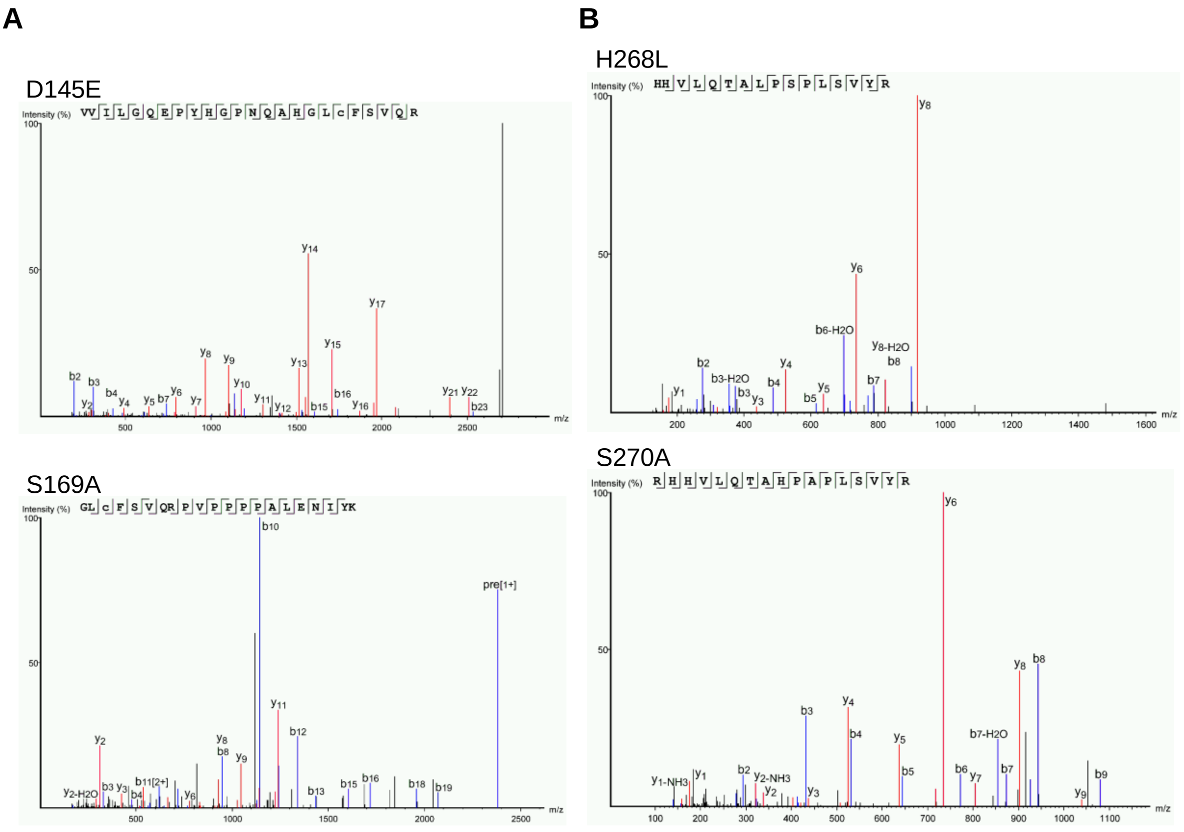

Supplementary Figure 6 | The amino acid replacements were confirmed by Q Exactive (LC-MS/MS) and Denovo sequencing. Proteins were produced as described in Materials and Methods (see **Figure 3A**). Gel pieces were subjected to in-gel reduction, alkylation and tryptic digestion using 6 ng/µl trypsin (V511A, Promega, Wisconsin, USA) [Shevchenko, A., Wilm, M., Vorm, O. and Mann, M. (1996) Anal Chem, 68, 850–858]. OMIX C18 tips (Varian, Palo Alto, CA, USA) were used for sample cleanup and concentration. Peptide mixtures containing 0.1% (v/v) formic acid were loaded onto a Thermo Fisher Scientific EASY-nLC 1000 system and EASY-Spray column (C18, 2 µm, 100 Å, 50 µm, 50 cm). Peptides were fractionated using a 2–100% (v/v) acetonitrile gradient in 0.1% (v/v) formic acid over 50 min at a flow rate of 200 nl/min. The separated peptides were analyzed using a Thermo Scientific Q Exactive mass spectrometer. Data was collected in data dependent mode using a Top10 method. The fragmentation spectra were searched by the PEAKS Studio 7 software (v. 7.0, Bioinformatics Solutions, Canada) using a human Swiss-Prot database, for mutations in the hUNG protein. The PEAKS software was used for peptide identification (Protein ID) through *de novo* sequencing assisted database searching. Peptide mass tolerances used in the search were 5 ppm, and fragment mass tolerance was 0.01 Da. The mutations in the proteins were identified using a peptide mutations and homology search and by using the SPIDER module in PEAKS.

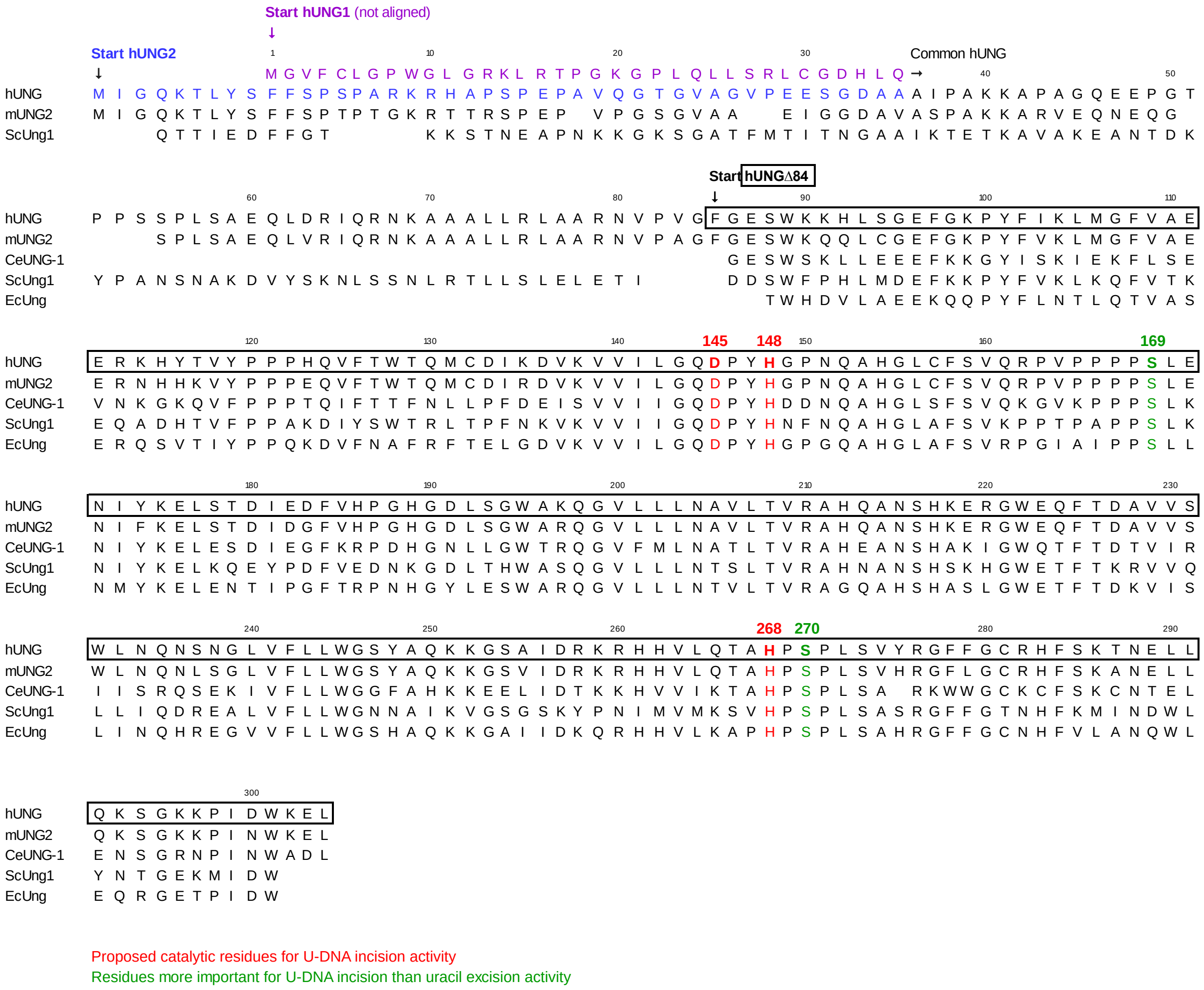

Supplementary Figure 7 | Alignment of the hUNG amino acid sequence with those of UNG enzymes of some model organisms. NCBI Reference Sequences in parentheses: hUNG (hUNG2, NP_550433.1), mUNG2 (*Mus musculus*, NP_001035781.1), CeUNG-1 (*Caenorhabditis elegans*, NP_499560.1), ScUng1 (*Saccharomyces cerevisiae*, NP_013691.1) and EcUng (NP_417075.1). See **Figures 2–4** for details about residues in bold.
